# Effects of a home-based palliative heart failure program on quality of life among the elderly: a clinical trial study

**DOI:** 10.1186/s12904-023-01245-x

**Published:** 2023-09-06

**Authors:** Mohammad Hossein Khajehpoor, Parvin Mangolian shahrbabaki, Esmat Nouhi

**Affiliations:** 1 Nursing Research Center, Kerman University of Medical. Sciences, Kerman, Iran; 2 Department of Critical Care Nursing, Nursing Research Center, Razi Faculty of Nursing and Midwifery, Kerman University of Medical Sciences, Kerman, Iran; 3 Department of Medical-Surgical Nursing, Razi Faculty of Nursing and Midwifery, Kerman University of Medical Sciences, Haft-Bagh Highway, PO Box 7716913555, Kerman, Iran

**Keywords:** Palliative Care, Home Care, Elderly, Heart Failure, Quality of Life

## Abstract

**Introduction:**

One of the frequent issues that lowers elderly people's quality of life is chronic heart failure, a progressive and life-limiting disease. The purpose of this study was to evaluate the effects of home-based palliative care (HBPC) on the quality of life of elderly patients with heart failure who received discharge orders from hospitals affiliated with Kerman University of Medical Sciences in 2022.

**Methods:**

One hundred heart failure patients were divided into two intervention and control groups for this randomized clinical trial study. The patients were then given the pre-test questionnaires, such as the demographic questionnaire and the Quality of Life Index (QLI) by Ferrans and Powers. The intervention group was given the home care plan. To measure the quality of life one month after the intervention, the quality of life questionnaire was lastly filled out by both groups following the last care session. Software called SPSS 22 was used to enter and analyze the patient data.

**Results:**

The mean age for the elderly in the intervention and in the control groups were 69.46 ± 11.61 and 66.14 ± 12.09 years, respectively. The palliative care program at home made a statistically significant difference in the quality of life and all of its components in the elderly with heart failure in the intervention group immediately after the intervention and one month after the intervention compared to before (*P* < 0.001). As a result, its scores improved compared to the stage before the intervention. Additionally, a significant difference between the quality of life score and all of its components between the intervention's immediate aftermath and one month later was noted (*P* < 0.05).

**Conclusion:**

Home-based palliative care has a positive effect on the quality of life for elderly people who have heart failure, making it a worthwhile intervention to enhance their quality of life.

**Trial registrations:**

(IRCT20211213053389N1). Date of registration: (19/02/2022).

## Introduction

A common aging disorder is heart failure (HF). HF is an advanced heart problem and one of the main causes of death and burden in many countries, particularly in low- and middle-income countries, mostly in the Eastern Mediterranean Region [1]. As an Eastern Mediterranean country, Iran has adopted a Western lifestyle. Such lifestyle changes, along with improved health services, have led to an improvement in life expectancy as well as an increase in the prevalence of non-communicable diseases including cardiovascular diseases (CVDs) [2, 3]. The first leading cause of mortality and a million disability-adjusted life years (DALYs) in Iran emanated from CVDs. Moreover, CVDs account for 46% of all deaths and 20%-23% of the burden of disease in Iran [1]. Compared to 2005, CVD-related DALYs are predicted to increase twofold by 2025 among Iranians aged ≥ 30 years. However, the prevalence among men will still be higher than among women; with a slightly smaller difference in 2025 [4]. HF prevalence ranges from 0.4% to 4.3% in the general population and from 2 to 20% in the elderly population over 75 years. The 1-year mortality rate of HF was 32% in Iran, showing a similar pattern to other countries [1]. Compared to other age groups, older adults with heart failure exhibit more physical symptoms like fatigue, shortness of breath, ankle or abdominal swelling, sleep problems, depression, and chest pain [5].

Numerous studies have demonstrated that people with heart disease experience various negative physical, psychological, emotional, and spiritual consequences. The combination of these symptoms restricts the patients' daily activities and makes it difficult for them to handle personal and social responsibilities, which lowers their quality of life. On the other hand, a decrease in quality of life is directly related to frequent hospitalizations and increased patient mortality. The World Health Organization (WHO) defines the quality of life as “an individual's perception of their position in life in the context of the culture and value systems in which they live and in relation to their goals, expectations, standards, and concerns” [6, 7]. Hospitalization and death rates are higher in older adults with heart failure who have a lower quality of life. Three million hospital admissions and numerous deaths caused by heart failure are reported each year in Europe [8]. In addition to eliminating the disease or alleviating symptoms, heart failure treatments aim to enhance the patient's quality of life in terms of their circumstances, social interactions, and relationships with others [9].

Palliative care has received support recently as part of the treatment of patients with heart failure [10]. WHO has defined palliative care as an approach to care that enhances the quality of life for people with life-limiting illnesses and their families by focusing on the prevention and relief of suffering through the early identification, assessment, and treatment of pain as well as by addressing physical, psychosocial, and spiritual needs [11]. According to Årestedt et al., expanding access to palliative care can help patients with heart failure receive better care during their final week of life [12]. Most patients feel more comfortable in their homes than in hospitals or nursing homes. In addition, HBPC enables family members to engage in the process [13]. In HBPC, family members directly participate in the care process. Therefore, the patient has easy access to care. Additionally, if necessary, the HBPC team can facilitate a prompt hospital referral [13, 14].

As the symptoms of these patients start affecting their quality of life, the burden of the disease increases, and thus, while the treatment for the disease continues, an advanced care plan is needed to increase the comfort of patients and relieve their symptoms [15]. PC interventions are an integral part of the care plan for heart failure patients, and the patients benefit more from the inclusion of the PC team in home-based healthcare institutions. Hospital-based PC is a more appropriate choice for patients who need more intensive symptom management or those who cannot cope with the family’s burden of care. Considering the benefits of HBPC and problems such as the aging population, the increase in HF rate, and limited and inadequate health resources, the integration of HBPC into health systems becomes important [16]. In Iran, providing care for patients with incurable diseases, especially those with end-stage diseases, is the responsibility of family members. Although all hospitals admit these patients and provide care to them, services specifically designed for these patients are very limited. Thus, these patients receive only routine hospital care and services provided to just any other patient. In this respect, there are a number of charitable or community palliative care centers scattered across the country. Palliative and supportive medical services are only provided in a few centers across the country [17]. For many healthcare staff and personnel, the COVID-19 lockdowns represented a dramatic shift inwards; venturing from home, previously innocuous, suddenly bore threat [18]. Even as the pandemic shows signs of subsiding in Iran, homebound patients with life-limiting illnesses still need home-based services to manage symptoms, care for their caregivers, and decrease burdensome costs. Considering the importance of improving the quality of life, end-of-life care, the vital role of nurses in this field, and also the small number of studies that have addressed palliative care in older adults with chronic heart failure in Iran, the present study aimed to examine the impact of home-based palliative care on quality of life in older adults with heart failure.

## Methodology

### Participants

This experimental study was carried out as a randomized controlled clinical trial with a 1:1 allocation ratio in the heart departments of hospitals affiliated with the Kerman University of Medical Sciences from December 2021 to September 2022. The number and accessibility of patients were the deciding factors in selecting these centers.

### Sampling

Elderly patients with heart failure who were discharged from hospitals affiliated with the Kerman University of Medical Sciences made up the study population. The Centers for Disease Control and World Health Organization defined aged populations as ≥ 65 chronological years in human studies. By definition, older adults are identified as individuals ≥ 65 [19]. Older adults with a confirmed diagnosis of chronic heart failure (class III or IV according to the American Heart Association's (NYHA) classification) [10] with the capacity for communication, the absence of speech or hearing issues [10, 20], the mental capacity to complete the questionnaire [21], the absence of a mental illness and the use of drugs that impair cognition [22, 23], full alertness at the time of the study [22], and the willingness to participate were candidates for this study. The exclusion criteria were unsteady physical and emergency conditions, such as a change in vital signs and the patient's inability to participate in the intervention sessions [10], failure to attend more than two sessions, and failure to correctly answer more than one-third of the questions.

### Randomization

**Fig. 1:**
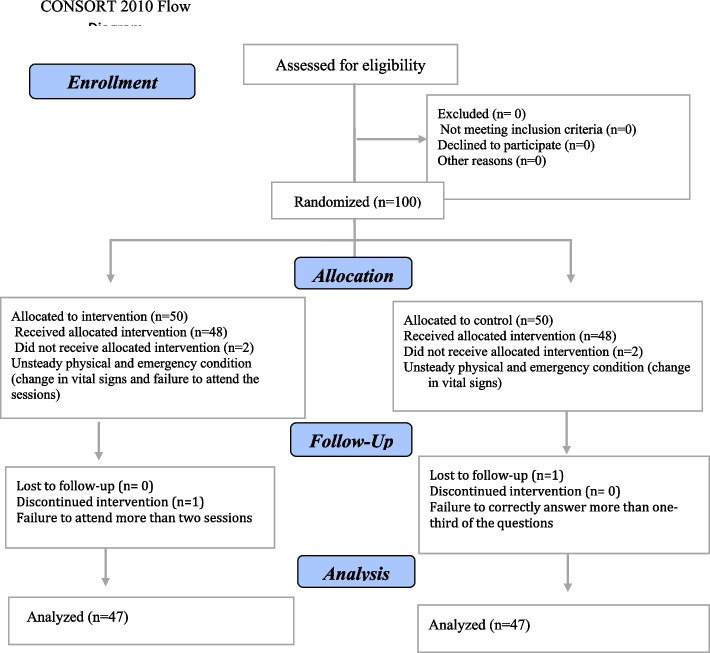
Explanation of sample size and sampling

In this study, 100 eligible patients were selected through convenience sampling and were then assigned to the control and intervention groups through permuted block randomization. The allocation sequence was performed using the free web system http://www.randomization.com. Thus, the number of subjects in each block was determined to be 5. Besides, letter A was considered for the control group and letter B for the test group. After confirming the allocation sequence created in the above system for 20 blocks, the allocation sequence was formed for 100 samples by combining the letters A and B. Finally, the cards specifying the blocks were placed inside a standard envelope for allocation concealment. Based on the number of eligible patients, an envelope was randomly selected by envelope shuffling to select the patients through random allocation. To reduce any possible bias in the randomization process, the random program was developed by a person who was not a member of the research team. Following the literature and a similar study [7], the sample size was estimated as 50 patients in each group based on the confidence coefficient (Z = 1.96), a power of 80%, and a 20% attrition rate. Six persons were excluded from the study for various reasons, including unstable physical and emergency conditions (acute changes in vital signs), failure to attend more than two sessions, and failure to adequately respond to the questions. Thus, the data for 94 persons were analyzed as displayed in CONSORT 2010 Flow Diagram (Fig. 1).

## Data collection

### Instruments

A three-part questionnaire was used to collect demographic and background information form and standard Ferrans and Powers' Quality of Life Index (QLI), to meet the study's objectives. The tools used in this study were translated into Persian, and the language of the used instruments was Persian.

The patient's age, gender, marital status, occupation, income, level of education, smoking status, length of the disease, number of prior heart-related hospitalizations, history of disease education, and other conditions like gastrointestinal disorders, diabetes, and blood pressure were measured using a demographic information questionnaire. Besides, the Quality of Life Index (QLI; Ferrans & Powers, 1984) was used to measure the patient’s quality of life.

### Quality of Life Index (QLI)

The cardiovascular patients' quality of life was assessed using the Quality of Life Index (QLI) developed by Ferrans and Powers (1984). This 70-item survey instrument uses a 6-item Likert scale to measure four factors underlying the QLI: health, socioeconomic, psychological/spiritual, and family connections (1 to 6). There are 35 items in each section, and items addressing the importance and satisfaction are similar. There are 15 items for measuring the health and functioning dimension, eight items for measuring the socioeconomic status, seven for measuring the psychological/spiritual dimension, and five items for measuring the family connections dimension. The scores were calculated using the Syntax program. To center the scale on zero and arrive at the QOL score, we subtracted 3.5 from the satisfaction response for each item, producing scores–2.5, -1.5, -0.5, + 0.5, + 1.5, and + 2.5. The scores of each option in the importance section were multiplied by the numbers obtained in the subsequent step. The outcomes from each option were then combined. The sum of the scores was then divided by the number of answered items, which ranged from + 15 to -15, to eliminate the impact of unanswered items. The range of scores was then established from 0 to 30, and the negative scores were eliminated by adding a fixed amount of + 15 to each of the calculated numbers.

Accordingly, a score of 0–9 indicated a poor quality of life, 10–19 indicated a moderately good quality of life, and 20–30 indicated a good quality of life. Cronbach's alpha for the QLI (total scale), which ranged from 0.73 to 0.99 in 48 studies, supported the internal consistency of the scale. Furthermore, the alpha ranges for the four subscales were as follows: 0.70 to 0.94 for the health and functioning subscale; 0.78 to 0.96 for the psychological/spiritual subscale; 0.71 to 0.92 for the socioeconomic subscale; and 0.63 to 0.92 for the family connections subscale [24, 25]. Borzou et al. (2014) compared individual and peer educational methods on the quality of life of heart failure patients, and obtained the reliability through Cronbach's alpha coefficient as 0.89 [26]. Moreover, ten professors at the University of Medical Sciences in Iran assessed the content validity of the questionnaire and confirmed its reliability with a Cronbach's alpha of 0.86 [27]. Khajali et al. (2022) used Cronbach’s alpha to evaluate the scale’s reliability, which was approved with a score of 0.86 [28]. Rafiei et al. (2014) designed a study in order to translate and validate the Persian version of the Quality of Life Index (QLI) questionnaire, The internal consistency for the global score was 0.934 indicating that all domains met the minimum reliability standard, the Cronbach's alpha ranged from 0.74 to 0.90 except for family subscale (α = 0.58). Test–retest reliability showed good results for global score (spearman's correlation = 0.89, ICC = 0.887) and for other domains except for family subscale (ICC = 0.255). The concomitant validity and construct validity revealed significant correlation between QLI with SF-36 questionnaire and Vaux questionnaire, respectively. Confirmatory factor analysis using EQS software also revealed that factor structure of the questionnaire in sample survey is repeated [29]. Dehesh et al. (2013) assessed convergent, discriminant, and construct validity of the questionnaire. construct validity of Ferrans and Powers is completely acceptable and their findings showed that the scaling success rates for discriminant validity of the all items was 100% (99/99), and this was true also for all subscales. Convergent validity was also 100% for all domains. Internal consistency reliability for the entire questionnaire and for all domains was supported by Cronbach’s alpha of 0.95, 0.88, 0.88, and 0.64 for HF, SE, PS, and FA, respectively, which were greater than 0.7, except for the family subscale. This study show that there is no cultural antithesis between original English and Persian versions in understanding the purposes of the items and that the Persian translation conducts the designers purposes and meanings to the responders perfectly [30]. In the present study we used Cronbach’s alpha to evaluate the scale’s reliability, which was approved with a score of 0.94 and content validity showed good result (CVI: 0/85).

## Intervention

The researcher visited the coronary care units (CCUs) of hospitals affiliated with the Kerman University of Medical Sciences to conduct the study and obtained the required approvals. Using the convenience sampling technique, the researcher divided the selected eligible patients into intervention and control groups. The patients were given the necessary information about the research objectives, and their written informed consent was obtained.

The older adults in the intervention group attended 8 individual sessions for one and a half hours twice a week. The home-based palliative heart failure program was conducted for one month with prior arrangements with the patients. The older adults in the control group only received routine care during this time. The intervention was delivered by one of the authors who was a senior cardiovascular nurse and was familiar with the interventions. The program focused on information about the disease, mental and physical aspects of self-care for old client and their families, an examination of coping strategies in stressful situations, and techniques to boost self-confidence (Table 1).

**Table 1:** The content of the HBPC program

**Session**	Summary of the sessions
**Session 1**	Introduction and recognition, explanation of the disease and symptoms and clinical complications of the disease, the introduction of self-care programs, stress management, depression, and anxiety management, emotional support, spiritual support, and improving communication
**Session 2**	Providing an introduction to the importance and necessity of self-care education in the mental and physical dimensions and monitoring symptoms
**Session 3**	Assessing the need for care of different body systems, examining the physical, psychological, and behavioral effects of older adults and their families
**Session 4**	Investigating self-care ability, stressful situations, social interactions and communication of older adults and life expectancy and the effects of death anxiety and family support and emotional and spiritual support
**Session 5**	Implementation of training programs for elderly clients and families based on self-care needs
**Session 6**	Examining coping methods in stressful situations, and ways to cope with stress and depression
**Session 7**	Strengthening self-confidence, self-esteem, coping with worry and anxiety and ineffectiveness
**Session 8**	Reviewing the past meetings, preparing the family to finish the group meetings, focusing on the implementation of self-care activities

During the intervention, the researcher performed the nursing assessment, education, and counseling and provided emotional support to patients. At the first visit, the researcher assessed the patients’ knowledge and abilities in self-management, their family support system, and their educational and counseling needs including symptoms and self-care of heart failure. An educational brochure was also provided to the patients. The brochure provided some instructions about heart failure, symptom management and monitoring, medication use, and the date of follow-up. The first face-to-face intervention session focused on enhancing participants’ knowledge about the effect of home-based palliative care on heart failure.

The researcher would provide emotional support to patients, encouraged them to express their feelings, listened actively to patients’ responses, reassured the patients that their feelings were normal, and supported them in taking small steps to resolve the problem. In addition, the researchers provided 24-h phone calls for the participants to help them solve their problems. Every week after 2 face-to-face session interventions, the researchers contacted the patients by phone at home to assess potential problems and to make an appointment for the next intervention. Finally, the follow-up evaluations conducted after the 8^th^ session enabled the researchers to provide continuous nursing care and mental support and, thus, enhance the participants’ ability to self-care. These subsequent follow-up phone calls reinforced the content of education and monitored the participants’ symptom management and progress.

The researcher completed the questionnaire for the intervention and control groups before the first session (before the intervention) and after the last session (after the intervention). The researcher re-completed the questionnaires for the intervention and control groups over the phone at the scheduled times after one month had passed (follow-up). The content of the intervention was constructed based on the guide for the care of patients with advanced chronic heart failure at home, which was compiled by the Secretariat of the Strategic Council for the Development of Health Guidelines of the Ministry of Health and Medical Education of Iran. In designing the intervention, the clinical guidelines for the management of chronic heart failure, which were compiled by the Department of Standardization and Compilation of Clinical Guidelines and the Office of Technology Evaluation, Standardization and Health Tariff of the Ministry of Health and Medical Education of Iran, were used. Also, the content of the intervention is based on the country's ethical guidelines on the subject of supportive palliative care in patients at the end of life, which were prepared by the Medical Ethics Department of the Endocrine and Metabolism Research Institute of Tehran University of Medical Sciences and supported by the World Health Organization Office in the Ministry of Health and Medical Education of Iran. Finally, the validity and caliber of the intervention were assessed by palliative care specialists at Kerman University of Medical Sciences.

## Data analysis

SPSS software version 22 was used to enter and save patient data. The data on the demographic and quantitative variables were summarized using absolute and relative frequency, mean, and standard deviation. The normality of the data was checked using the Kolmogorov–Smirnov test. The data were analyzed with the chi-square test, independent samples t-test, and repeated measures ANOVA. The impact of each independent variable on the outcome variables was assessed using multivariate linear regression analysis with a test power of 0.80 and a significance level of 0.05.

### Ethical Considerations

This study was conducted following the ethical code IR.KMU.REC.1400.633 issued by Kerman University of Medical Sciences and the arrangements made with the officials at Razi Nursing and Midwifery College and hospital management. Some instructions were provided to the participants about the objectives of the study, voluntary entrance to and withdrawal from the study, and the application of the results. If required, the authorities were also given access to the findings. The study was conducted based on the legal, ethical, and professional norms of the community. The protocol for this study was registered under number IRCT20211213053389N1 on 19/02/2022.

## Results

The participants’ age ranged from 60 to 80 years. The mean age of the patients (*n* = 47) in the intervention group was 69.46 ± 11.61 years and that of the patients (*n* = 47) in the control group was 66.14 ± 12.09 years (Table 2). As shown in Table 2, there was no statistically significant difference between the two groups in terms of demographic variables (*P* > 0.05). Based on the Bonferroni correction, pairwise comparisons of the quality-of-life score and its components were performed for the intervention and control groups as reported in Tables 3 and 5.

**Table 2:** Comparison of demographic information score of heart failure patients

**Variable**	**Group**					
	**Intervention**		**Control**		**Statistical analysis**	***P ***value
	**Mean**	**SD**	**Mean**	**SD**		
Age	69.46	11.61	66.14	12.09	t-test = 1.357^b^	0.178
	***n***	**%**	***n***	**%**	**Statistical analysis**	***P*** value
Sex						
Female	20	42.6	21	55.3	χ^2^ = 1.54^a^	0.216
Male	27	57.4	26	44.7		
Marital status						
Single	14	29.8	7	14.9	χ^2^ = 3.01^a^	0.083
Married	33	70.2	40	85.1		
Education						
Illiterate	15	31.9	24	51.1	χ^2^ = 3.55^a^	0.169
Middle school	18	38.3	13	27.7		
Diploma	13	27.7	9	19.1		
Bachelor and higher	1	2.1	1	2.1		
Employment status						
Self-employed job	10	21.3	9	19.1	χ^2^ = 4.37^a^	0.224
Retired	17	36.2	9	19.1		
Unemployed	5	10.6	9	19.1		
Housekeeper	15	31.9	20	42.7		
Smoking						
Yes	18	38.3	19	40.4	χ^2^ = 0.05^a^	0.833
No	29	61.7	28	59.6		
History of hospitalization						
Yes	41	82.2	42	89.4	χ^2^ = 0.11^a^	0.748
No	6	12.8	5	10.6		
Having another chronic disease						
Yes	39	83	34	72.3	χ^2^ = 1.53^a^	0.216
No	8	17	13	27.7		
Duration of illness (years)						
< 1	10	21.3	15	31.9	χ^2^ = 7.70^a^	0.053
1–5	18	38.3	25	53.2		
5–10	13	27.7	5	10.6		
> 10	6	12.8	2	4.3		
History of education about the disease						
Yes	30	63.8	26	44.7	χ^2^ = 3.47^a^	0.062
No	17	36.2	21	55.3		

*n* Frequency

% Percent of frequency

^a^Chi-squared test

^b^Independent t-test

**Table 3:** Pairwise comparisons of the QOL score and its components in the research stages in the intervention group

**Variable**	**Stage**		**Group**	
			**Intervention**	
			**Meandifference**	*P *value^*^
	Before the intervention	Immediately after the intervention	-6.570	< 0.001^**^
Quality of Life		One month after the intervention	-8.967	< 0.001^**^
	Immediately after the intervention	One month after the intervention	-2.397	< 0.001^**^
	Before the intervention	Immediately after the intervention	-7.368	< 0.001^**^
Health and functioning		One month after the intervention	-9.294	< 0.001^**^
	Immediately after the intervention	One month after the intervention	-1.836	< 0.001^**^
	Before the intervention	Immediately after the intervention	-5.406	< 0.001^**^
Socioeconomic		One month after the intervention	-8.287	< 0.001^**^
	Immediately after the intervention	One month after the intervention	-2.882	< 0.001^**^
	Before the intervention	Immediately after the intervention	-7.280	< 0.001^**^
Psychological/Spiritual		One month after the intervention	-9.699	< 0.001^**^
	Immediately after the intervention	One month after the intervention	-2.419	< 0.001^**^
	Before the intervention	Immediately after the intervention	-5.045	< 0.001^**^
Family Connections		One month after the intervention	-8.317	< 0.001^**^
	Immediately after the intervention	One month after the intervention	-3.272	< 0.001^**^

^*^_Bonferroni: adjustment for multiple comparisons_

^**^_Statistically significant difference according to Bonferroni correction_

Before HBPC, there was no statistically significant difference between the two groups in the quality of life and its subscales. A significant difference was found between the two groups in the quality of life and its subscales immediately after HBPC and one month later (*P* < 0.001)(Table 4).

**Table 4:** Comparison of the QOL score and its components between intervention and control groups before, immediately after, and one month after HBPC

**Variable**	**Group**						
	**Intervention**		**Control**		**Independent t-test**		***P *value**
	**Mean**	**SD**	**Mean**	**SD**			
**Quality of life**							
Before the intervention	14.70	1.20	14.48	1.19	0.91		0.366
Immediately after intervention	21.27	2.12	15.33	1.10	17.04		< 0.001
One month after the intervention	23.67	2.14	15.60	1.20	22.46		< 0.001
**Health and functioning**							
Before the intervention	14.06	1.19	13.95	1.25	0.43		0.688
Immediately after intervention	21.43	2.10	15.46	1.58	15.53		< 0.001
One month after the intervention	23.26	2.19	15.89	1.71	15.89		< 0.001
**Socioeconomic**							
Before the intervention	14.97	1.36	14.68	1.28	1.08		0.285
Immediately after intervention	20.38	3.29	14.82	1.28	10.76		< 0.001
One month after the intervention	23.26	2.99	14.96	1.29	17.42		< 0.001
**psychological/spiritual**							
Before the intervention	14.71	1.81	14.21	1.59	1.40		0.165
Immediately after intervention	21.99	2.42	15.45	1.69	15.16		< 0.001
One month after the intervention	24.41	2.35	15.68	1.88	19.83		< 0.001
**Family Connections**							
Before the intervention	16.21	1.92	16.11	1.63	0.25		0.804
Immediately	21.25	3.61	15.56	1.70	9.76		< 0.001
One month after the intervention	24.52	2.90	15.61	1.71	18.11		< 0.001
**Source of difference**	**Sum of squares**		**df**	**F**		***P *value**	**Eta2**
Time	1283.407		2	467.55		< 0.001	0.836
Group^a^ time interaction	774.496		2	282.15		< 0.001	0.754
Group	1590.012		1	345.46		< 0.001	0.790
Error	423.439		92				

^a^_Bonferroni: adjustment for multiple comparisons_

Compared to the pre-HBPC program, the quality of life and its subscales among the heart failure patients in the intervention group were significantly higher immediately after HBPC and one month later (*P* < 0.001). Additionally, a significant difference was found between the QOL score and its constituent parts one month after the intervention and right after the intervention, confirming the retention of the effectiveness of palliative care at home (Table 3).

Moreover, there were statistically significant differences in the QOL score and its components (aside from economic, social, and family connections) in older adults with heart failure in the control group immediately following the intervention and one month later compared to before the intervention (*P* > 0.05). As a result, the QOL scores improved in comparison to the corresponding scores before the intervention (Table 5).

**Table 5:** Pairwise comparisons of the QOL score and its components in the research stages in the control group

**Variable**	**Stage**			
			**Control**	
			**Meandifference**	*P *value^*^
	Before the intervention	Immediately after the intervention	-0.849	0.005^**^
Quality of Life		One month after the intervention	-1.118	< 0.001^**^
	Immediately after the intervention	One month after the intervention	-0.269	0.295^***^
	Before the intervention	Immediately after the intervention	-1.509	< 0.001^**^
Health and functioning		One month after the intervention	-1.938	< 0.001^**^
	Immediately after the intervention	One month after the intervention	-0.429	0.016^**^
	Before the intervention	Immediately after the intervention	-0.144	1^***^
Socioeconomic		One month after the intervention	-0.285	1^***^
	Immediately after the intervention	One month after the intervention	-0.141	1^***^
	Before the intervention	Immediately after the intervention	-1.242	0.002^**^
Psychological /Spiritual		One month after the intervention	-1.470	< 0.001^**^
	Immediately after the intervention	One month after the intervention	-0.288	0.850^***^
	Before the intervention	Immediately after the intervention	0.555	0.544^***^
Family Connections		One month after the intervention	0.504	0.629^***^
	Immediately after the intervention	One month after the intervention	-0.051	1^***^

^*^_Bonferroni: adjustment for multiple comparisons_

^**^_Statistically significant difference according to Bonferroni correction_

^***^_No_ _Statistically significant difference according to Bonferroni correction_

## Discussion

The present study examined the effectiveness of HBPC in the quality of life of older adults with heart failure. The findings showed no significant difference in the pre-intervention QOL scores between the intervention and control groups. The home-based palliative care program significantly improved the quality of life and all its components for the patients in the intervention group immediately after the intervention compared to the pre-intervention phase. However, there was significant difference in the QOL scores for the patients in the control group but these changes were less than the intervention group. These findings were in line with the results reported by Hosseini et al. [31] and Brännström et al. [32]. They demonstrated that patients with heart failure who received palliative care at home experienced an immediate improvement in their quality of life. In the control group, the quality and duration of routine care has not been evaluated, and the improvement in routine care just before the start of the trial cannot be ignored. The control group received routine care that included some primary palliative care (PPC), defined as the provision of some element of palliative care (e.g., primary symptomatic treatment, proactive care planning, or goal setting). Although, both specialty palliative care (SPC) in intervention group and routine care in control group were associated with improvements in outcomes, particularly quality of life, among patients with HF, there were notable differences in types of outcomes measured between SPC and PPC interventions (Tables 3 and 5). Compared to PPC, SPC interventions were more comprehensive and in terms of elements of palliative care, PPC were less likely to involve structural or physical aspects of care than SPC interventions [33]. Notably, no PPC interventions in this study addressed socioeconomic and cultural aspects of care, an important part of palliative care when considering cultural preferences for treatment, especially given the differences in palliative care. One of the main differences from routine care is that the HBPC team takes more comprehensive care of the person, taking into consideration symptoms and signs related to accompanying co-morbidities, such as stroke, renal dysfunction, pulmonary disorders, anemia, and even cancer and our approach was for HF management by integrating specialized palliative home care and heart failure care. The team is responsible for the total care of the patient. Assessment of symptoms, quality of life, and risk of pressure ulcers, falls, and malnutrition is ongoing. This does not mean that the patient's doctor or other professionals are held accountable, but rather that they are called upon to cooperate. HBPC program is also organized in close cooperation with out-of-hours palliative advanced home care. This team knows exactly the identity of patients and how to take calls. It should be noted that one of the main differences between the two groups was the nurse visits at the patient's home, which were substantially and significantly more frequent in the HBPC group compared with the control group. This made it possible to carry out the structured PC at home [32]. Also, the possibility for the patients to reach the personnel easily by phone and that treatment often could take place at the patient's home instead of them being admitted to hospital may have contributed to the achieved results. Therefore, the lack of a significant statistical difference in socioeconomic and family connections in the control group may be attributed to the need for a longer follow-up and supportive and palliative care for patients with chronic diseases and lack of attention to socioeconomic and cultural aspects of PC. Supportive and palliative services guarantee that part of the care helps the patient to have a better quality of life.

In line with the findings reported by Wong et al. [34], the data in the present study showed a significant difference in the health and functioning of older adults with heart failure between the group receiving palliative care at home and the control right after the intervention. However, these data were contrary to the findings reported by Bahadur et al. [35]. Previous studies have addressed patients with heart failure in all age groups whereas the current study concentrated on older adults. These age-related differences may account for the discrepancy in the results reported in the literature and the present study. The findings also revealed significant differences in the socioeconomic, psychological/spiritual, and family Connections factors between the group receiving palliative care at home and the control group right after the intervention. This result was in line with the findings reported by Bahadur et al. [35]. Following the findings in the present study, Yee Man Ng and Wong showed that the quality of life of heart failure patients improved after receiving palliative care at home [36].

The results revealed that the group receiving palliative care at home and the control group differed in terms of the quality of life one month after the intervention, as reported by Bahadur et al. [35]. Furthermore, a significant difference was found in the quality of life and its components one month after the intervention and right after the intervention in the intervention group, confirming the long-term effect of palliative care at home, as evident in studies by Reiser et al. [37] and Khalili et al. [38].

In addition, Greener et al. discovered that palliative care can enhance patients' quality of life and lessen their heart failure symptoms [39]. Isenberg et al. revealed that even minimal use of this care lowers hospital mortality and enhances the experience of dying [40]. They also compared people who received and did not receive HBPC services in the previous three months. A pilot study revealed that HBPC for patients with advanced CHF may raise the likelihood of passing away at the selected location while lowering hospital admissions [41]. The quality of life of heart failure patients improved in the study by Yee Man Ng and Wong after 12 weeks of receiving HBPC [36], as evident in the current study. HBPC and structured home visits with the assistance of a multidisciplinary team would improve symptom control and quality of life in patients with heart failure [34, 42]. Following these observations, it can be argued that older adults with heart failure require formal and structured palliative care programs that place a strong emphasis on all facets of life. Palliative care programs give these patients end-of-life care in addition to enhancing their quality of life and minimizing their symptoms throughout their lives. Even though these programs may not be able to lengthen the patients' lives, they still give them a high-quality and fulfilling life, even if they are only short programs [43]. These facts and findings provide substantial evidence supporting the need for a health care delivery system that more closely reflects the needs of chronically ill patients. Instead of care that is only curative until death, a new continuum must be created that provides a blended model of care.

## Conclusion

The findings showed that patients with heart failure who received HBPC had a higher quality of life. Improving the quality of life of elderly heart failure patients requires significant palliative care in addition to symptom management. Hence, palliative care should be incorporated into the treatment of this particular group of patients because it places a strong emphasis on comprehensive care. We recommend further exploration of the role of HBPC for diverse populations and its impact on health equity. Additionally, while the majority of studies have addressed older adults, no study has examined partnerships between geriatric medicine and palliative care specialists. Future research may clarify whether multidisciplinary collaboration across palliative care, primary care, and geriatric medicine offers unique benefits for home-bound patients. One area for strengthening palliative care trials is explicitly basing them in behavioral intervention and theories of palliative care delivery and studies should be designed to identify the active ingredients of effective palliative care and their causal pathways in impacting outcomes.

### Limitations

One of the limitations of this study was that the patients refused to attend the sessions due to fatigue brought on by heart failure symptoms. By placing restrictions on participation in the study, attempting to win patients' trust and cooperation, and encouraging patients to attend intervention sessions at times that did not conflict with their routines and treatment, it was attempted to solve the problem.

## Data Availability

The datasets used and/or analyzed during the current study are available from the corresponding author upon reasonable request.

## Abbreviations

HBPC: Home-based palliative care
PC: Palliative care
CVD: Cardiovascular disease
